# Assessing balance in people with bilateral vestibulopathy using the Mini-Balance Evaluation Systems Test (Mini-BESTest): feasibility and comparison with healthy control data

**DOI:** 10.1007/s00415-023-11795-y

**Published:** 2023-06-03

**Authors:** Meichan Zhu, Lisa van Stiphout, Mustafa Karabulut, Angélica Pérez Fornos, Nils Guinand, Kenneth Meijer, Raymond van de Berg, Christopher McCrum

**Affiliations:** 1 Division of Balance Disorders, Department of Otorhinolaryngology and Head and Neck Surgery, School for Mental Health and Neuroscience, Maastricht University Medical Center, Maastricht, The Netherlands; 2 Department of Nutrition and Movement Sciences, NUTRIM School of Nutrition and Translational Research in Metabolism, Maastricht University, Maastricht, The Netherlands; 3 Department of Otorhinolaryngology, Guangzhou Twelfth People’s Hospital (Guangzhou Otolarynology-Head and Neck Surgery Hospital), No. 1 Tianqiang Road, Tianhe District, Guangzhou, 510620 Guangdong China; 4 Service of Otorhinolaryngology and Head and Neck Surgery, Department of Clinical Neurosciences, Geneva University Hospitals, Geneva, Switzerland

**Keywords:** Balance, Mini-Balance Evaluation Systems Test, Bilateral vestibulopathy

## Abstract

**Objectives:**

Bilateral vestibulopathy (BVP) leads to unsteadiness when walking, which worsens in darkness or on uneven ground, as well as falls. Since simple balance tests struggle to distinguish between BVP and healthy participants, we aimed (1) to test if the Mini-BESTest is feasible in BVP, (2) how people with BVP perform on the Mini-BESTest and (3) to compare these scores with healthy reference data.

**Methods:**

Fifty participants with BVP completed the Mini-BESTest. 12-month falls incidence was obtained by questionnaire. To compare the overall and sub-scores between our participants with BVP and those of healthy participants from the literature (*n* = 327; obtained via PubMed searches), Mann–Whitney *U* tests were used. Sub scores within the BVP group were also compared. Spearman correlations were used to investigate the relationships between Mini-BESTest score and age.

**Results:**

No floor or ceiling effects were observed. Participants with BVP had significantly lower Mini-BESTest total scores than the healthy group. Anticipatory, reactive postural control and sensory orientation sub scores of the Mini-BESTest were significantly lower in BVP, while dynamic gait sub scores were not significantly different. A stronger negative correlation between age and Mini-BESTest total score was found in BVP than in the healthy group. Scores did not differ between patients with different falls history.

**Conclusion:**

The Mini-BESTest is feasible in BVP. Our results confirm the commonly reported balance deficits in BVP. The stronger negative association between age and balance in BVP might reflect the age-related decline in the remaining sensory systems with which people with BVP compensate.

**Supplementary Information:**

The online version contains supplementary material available at 10.1007/s00415-023-11795-y.

## Introduction

Bilateral vestibulopathy (BVP) is characterized by bilateral hypofunction of the vestibular organ or nerves which is diagnosed using the criteria reported by the Barany Society [1]. The symptoms of BVP include oscillopsia and postural instability. Importantly, BVP has many different aetiologies with many different disease presentations [2]. About 30–40% of people with BVP suffer from oscillopsia during head motion, due to reduced or absent vestibular-ocular reflex (VOR) [3]. In addition, people with BVP have a higher risk of falls [4], increased gait variability [5, 6] and often report difficulty and instability while walking in dimly lit environments and on uneven ground [7–9]. Consequently, avoidance of falls and reduction in mobility may occur and negatively impact societal participation, which may be linked with the increase in depression and reduced quality of life in people with vestibular disorders [10–15].

Deficient vestibular function may be defined using video head impulse testing (vHITs), caloric testing and torsion swing test and evaluation of dynamic visual acuity (DVA), cervical and ocular vestibular-evoked myogenic potentials for otolith function and Romberg testing for balance [1, 16, 17]. However, McCrum et al. [6] found no clear correlation between various gait variability parameters and the results of the caloric, vHIT and DVA tests, indicating that specific, objective assessment of balance and gait may be required in BVP to gain a better picture of a patient’s deficits.

Regarding the increasing falls risk in BVP, the data on exactly how falls occur is limited [4, 8, 9, 12, 18, 19]. People with BVP compensate with a combination of the remaining proprioceptive and visual function. As such, their balance might not be sufficiently examined by tasks which use zero or only a single sensory perturbation [20]. Tools such as the Berg Balance Scale (BBS), the Timed “Up & Go” Test (TUG) and the Romberg test, which is recommended by the Barany Society [1], may therefore, not be suitable [20]. Herssens et al. [20] reported that few studies have concentrated on the balance abilities of people with BVP during walking. This is critical since evaluation of the systems underlying balance deficits and falls in BVP is needed to identify and intervene on those deficits [20]. As Horak et al. [21] outlined, balance (or postural control) is a complex skill based on the interaction of dynamic sensorimotor processes, including active alignment of the head and body (with respect to gravity, support surfaces, the visual surround and internal references), integration and weighting of sensory information from somatosensory, vestibular and visual systems, with each of these broad factors being weighted according to the current task, goals and environment. For this reason, Herssens et al. [20] recommended that further research on the Balance Evaluation Systems Test [22] in BVP is needed. The refined Mini-Balance Evaluation Systems Test (Mini-BESTest) assesses multiple dynamic balance tasks related to four subcomponents of balance (anticipatory postural adjustments, reactive postural control, sensory orientation, and dynamic gait) [23]. However, its feasibility and ability to assess the specific balance deficits reported in people with BVP has yet to be evaluated.

To address the current gap in research and practice, this study investigated balance function in people with BVP using the Mini-BESTest. We aimed to determine whether the Mini-BESTest can be used with people with BVP (in terms of completion, floor and ceiling effects) and to explore how people with BVP perform in terms of their overall score and their subcomponent scores and if these reveal consistent balance deficits within this group (are specific subcomponents of balance more severely affected in BVP?) and in relation to healthy reference data from the literature (in terms of total score and if subscores focused on sensory function show larger differences). We expected that people with BVP would record lower overall scores than healthy participants and that subcomponents more clearly associated with sensory function would reveal larger deficits.

## Methods

### Participants

As part of a large prospective study, participants were recruited from Maastricht University Medical Center (MUMC+) and other tertiary referral centres in the Netherlands. Within the MUMC+, all patients diagnosed with BVP at the outpatient clinic of the Department of Otorhinolaryngology were asked to participate in the study in the period from June 2021 to June 2022. Ethical approval was granted by the azM/UM Medical Ethical Committee (METC: NL72200.068.19). Before participating in the study, each participant provided written informed consent. In the current manuscript, we report and analyse the Dutch version Mini-BESTest results from the larger project. Since this was a secondary analysis of the larger trial, no a priori sample size calculation was performed for the current analysis. Sensitivity power analyses are reported in the data analysis section below.

Fifty-three people previously diagnosed with BVP participated in this research. Recruited participants were formerly diagnosed with BVP according to Barany Society BVP diagnostic criteria [1]. On the testing day of the larger project, the diagnosis procedure was repeated, at which point two people were excluded because they no longer had BVP according to the criteria, leaving 51 participants. In the current study, 50 people with BVP are included because one participant did not perform the Mini-BESTest. The exclusion criteria were: a history of other neurological disorders; age under 18 and unable to discontinue vestibulo-suppressive medication. In addition to the procedures described below, participants provided information on their falls incidence in the previous 12 months by completing a questionnaire based on the recommendations of Lamb et al. [24] and Lord et al. [25] translated into Dutch (materials available at McCrum [26]), that led with the question “In the past year, have you had any fall including a slip or trip in which you lost your balance and landed on the floor or ground or lower level?”.

### Mini-BESTest Assessment

The Mini-BESTest was used in this study to evaluate the participants’ balance [23]. The Mini-BESTest evaluates balance tasks associated with various components of balance, namely anticipatory, reactive postural control, sensory orientation and dynamic gait. It comprises of 14 items and each item is scored on a 3-level ordinal scale from 0 (severe = unable to perform), 1 (moderate) and 2 (normal performance) with a maximum score of 28. The Mini-BESTest items are shown in Table 1. The Mini-BESTest was administered and rated by a trained member of the research team. We followed the full standardised equipment, testing and assessment procedures, which can be found at https://www.bestest.us/ with additional information in the scientific publication [23]. The test was performed as the first assessment in the morning of the larger project’s testing day to ensure mental or physical fatigue would not affect the results.

**Table 1:** Sections and items of the Mini-BESTest [23]

Section	Item
Anticipatory	1. Sit to stand 2. Rise to toes 3. Stand on one leg
Reactive postural control	4. Compensatory stepping correction—forward 5. Compensatory stepping correction—backward 6. Compensatory stepping correction—lateral
Sensory orientation	7. Stance (feet together); eyes open, firm surface 8. Stance (feet together); eyes closed, foam surface 9. Incline—eyes closed
Dynamic gait	10. Change in gait speed 11. Walk with head turns—horizontal 12. Walk with pivot turns 13. Step over obstacles 14. Timed Up and Go with dual task

### Healthy reference data

To compare our results from participants with BVP to representative healthy control data, 21 articles from the literature were identified (see the preprint mentioned in the Acknowledgements for full details). From these, we obtained complete individual data for Mini-BESTest total and sub scores from 190 healthy participants in four articles [27–30] and individual Mini-BESTest total scores from an additional 137 healthy participants in four articles [31–34] These data were combined to form healthy control reference data groups for our analyses (see below). The available group-level data in the remaining 11 articles [35–45] were used for additional visual comparison.

### Data analysis

Due to the current study conducting secondary analyses of a larger project, no a priori sample size calculation was performed. To indicate the statistical power of the current analyses, we performed sensitivity power analyses for the current sample sizes and tests with *α* = 0.05 and *β* = 0.2 using G*Power (Version 3.1.9.4) [46] which revealed effect sizes of 0.42 for Wilcoxon signed rank tests (*N* = 49), 0.44 (*N* = 50 vs. *n* = 327) and 0.46 (*N* = 50 vs. *N* = 190) for Mann–Whitney tests.

To assess Mini-BESTest sub score differences, a Friedman test within the BVP group was performed with sub score as the repeated measures factor, with post hoc Bonferroni-adjusted Wilcoxon signed rank tests for pairwise comparisons (note that to account for the different number of items in the sub scores, the sub scores were divided by the number of items before these analyses). To compare the total scores and sub scores between our participants with BVP and the obtained individual data of healthy control participants from the literature, Mann–Whitney *U* tests were used. The statistical analyses were conducted in Jamovi version 2.2.5 [47]. In addition to the statistical tests, the BVP data were visually compared to the summary values from all other studies obtained from the literature using GraphPad Prism 9 (GraphPad Software, San Diego, California USA, http://www.graphpad.com). All quantitative variables are expressed as mean ± SD, unless stated otherwise. Effect sizes for non-parametric tests were calculated as Cohen’s *d* according to Fritz et al. [48].

## Results

### Participant characteristics

We collected 49 total scores, 49 reactive postural control sub scores and 50 of all other sub scores from our participants with BVP. For one participant, the reactive postural control part of the Mini-BESTest could not be completed for practical reasons and therefore, only the other three sub scores were included in the analyses for this participant. Regarding the healthy control reference data, 327 healthy participants from eight articles [27–34] formed the control group for the Mini-BESTest total score and 190 healthy participants from four articles [27–30] formed the control group for the Mini-BESTest sub scores. Note that two of the contacted authors provided data for more participants than were reported in the identified study [33, 34]. For studies evaluating participants twice or using two assessors to score the performance, we always used the data of the first trial or first assessor reported. One participant in the dataset of Nakhostin-Ansari et al. [30] was excluded from the current analysis due to age (16 years old). Healthy participants were selected from the large public dataset of Santos, Duarte [28] based on the health and clinical information provided to form a younger adult group and middle to older aged adult group. The summary data obtained from the remaining 11 articles [35–45] found with the PubMed search were collated for visual comparison. Demographic data are illustrated in Table 2. The mean ages and sex distributions between the BVP and healthy groups were not statistically significantly different (Table 2). Significant group differences were found in height, body weight and body mass index (BMI) between two groups (Table 2).

**Table 2:** Demographic data of the participants with BVP and Healthy Control Reference Groups

	BVP	*N*	Healthy control	*N*	Statistical comparison
Age, years	57.56 ± 12.39	50	60.07 ± 17.40	305*	*t* test *p* = *0.328*
Male, *n* (%)	28 (56.0)	50	165 (54.1)	305*	Chi square *χ*^2^ = 0.06, *p* = 0.802
Height, cm	172.46 ± 8.92	50	164.79 ± 9.20	221*	*t* test *p* < 0.001
Body weight, kg	85.44 ± 21.47	50	66.19 ± 12.90	221*	*t* test *p* < 0.001
BMI	28.55 ± 6.26	50	24.30 ± 3.85	221*	*t* test *p* < 0.001

Mean ± standard deviation unless otherwise indicated

*Demographic data was not obtained from all studies (*n* = 327)

### Mini-BESTest feasibility

Regarding the Mini-BESTest completion and scores, one participant with BVP scored the maximum (28) and one participant with BVP scored the minimum score (0). We, therefore, confirm that in this sample, no floor or ceiling effects were observed. Other than the reasons for exclusion mentioned above, no complications or concerning situation arose during the testing.

### Mini-BESTest total scores

**Fig. 1:**
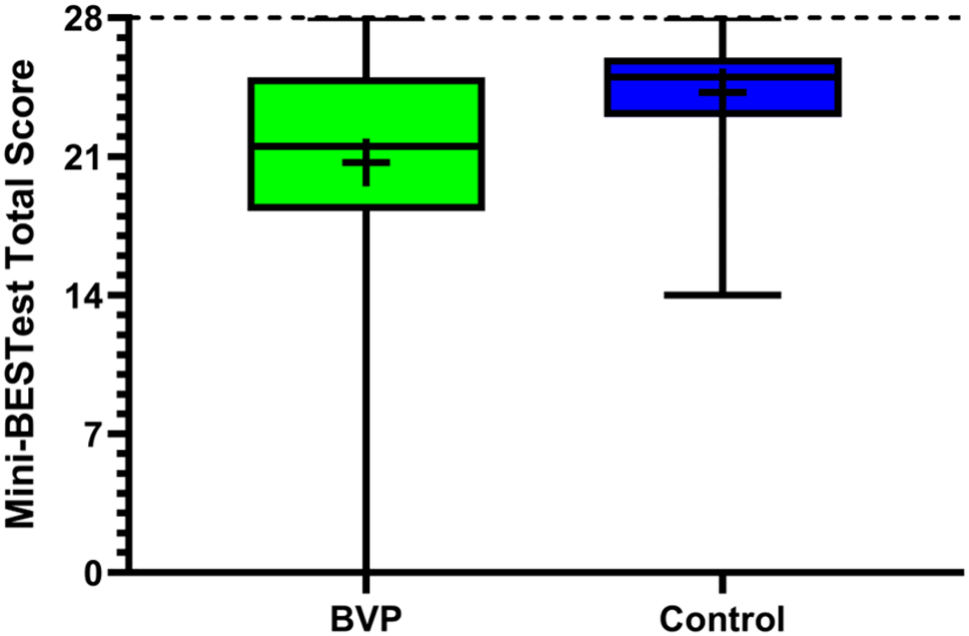
Mini-BESTest total scores in our BVP and healthy control groups. The boxplots indicate the medians, interquartile range and minimum and maximum values, with the cross indicating the mean values. Maximum score possible indicated by the dotted line

**Fig. 2:**
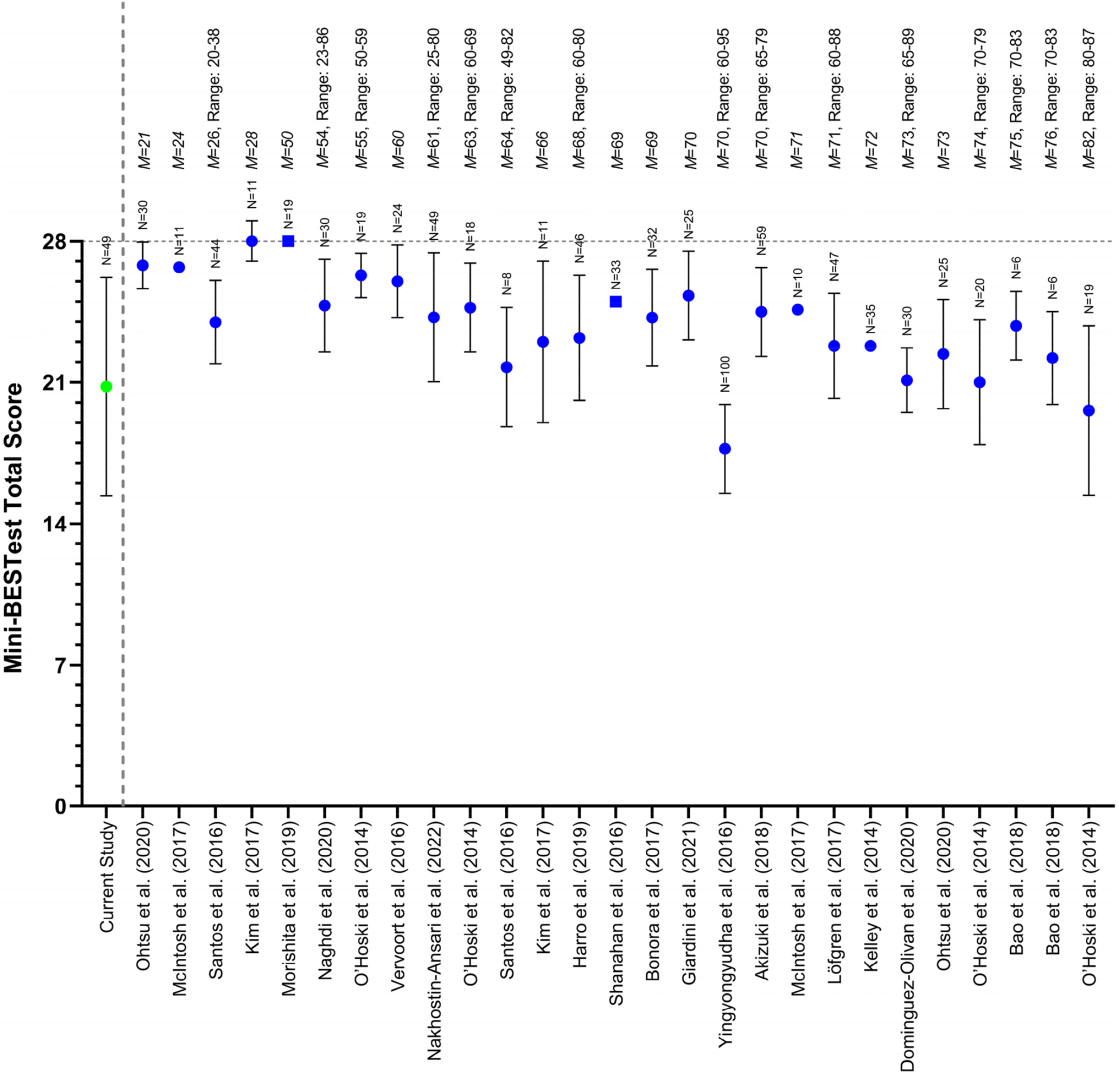
Mini-BESTest total scores (circles: mean ± standard deviation; squares: medians) for the current participants with BVP and healthy reference values found in the literature (left to right is youngest to oldest mean age). Values are shown here as originally reported, meaning that different rounding conventions have been used

A Mann–Whitney U test revealed that Mini-BESTest total scores were significantly lower than those observed in the healthy control reference data (Fig. 1; *U*(*N*_BVP_ = 49, *N*_Healthy_ = 327) = 4564.00, *p* < 0.001, *d* = 0.52) and this effect size exceeded what our sensitivity power analysis determined we could detect. Participants with BVP scored 20.78 ± 5.41 while the healthy controls scored 24.24 ± 2.72. Mean and standard deviation values from the current study and the identified studies in the literature are shown in Fig. 2, which seem to agree with our statistical analysis, in that the majority of studies on healthy adults report higher mean Mini-BESTest total scores than our BVP group.

### Mini-BESTest sub scores

**Fig. 3:**
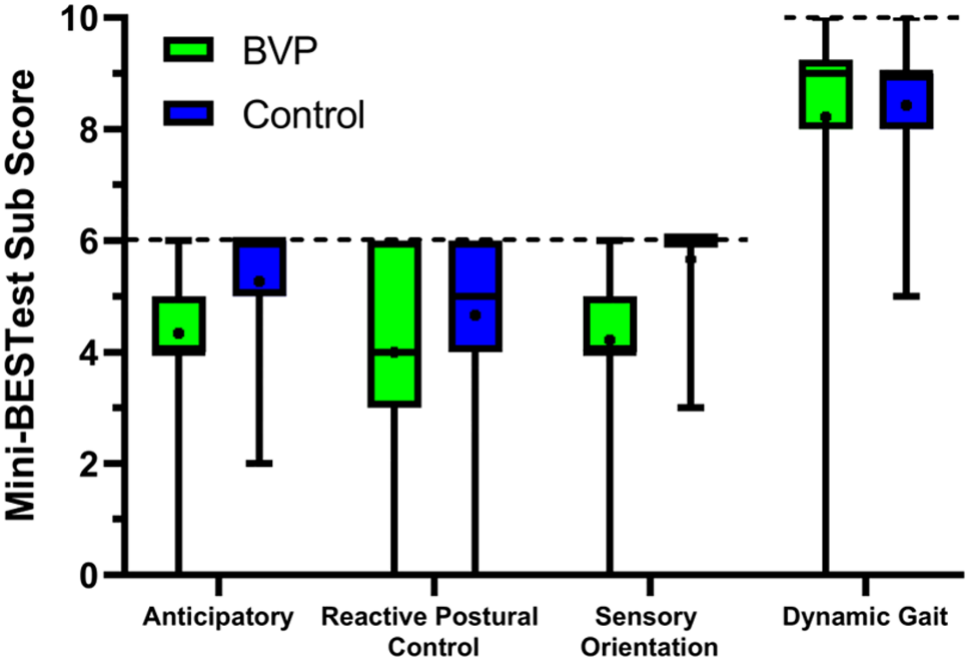
Mini-BESTest sub scores in our BVP and healthy control groups. The boxplots indicate the medians, interquartile range and minimum and maximum values, with the points indicating the mean values. Maximum score possible indicated by the dotted lines

**Fig. 4:**
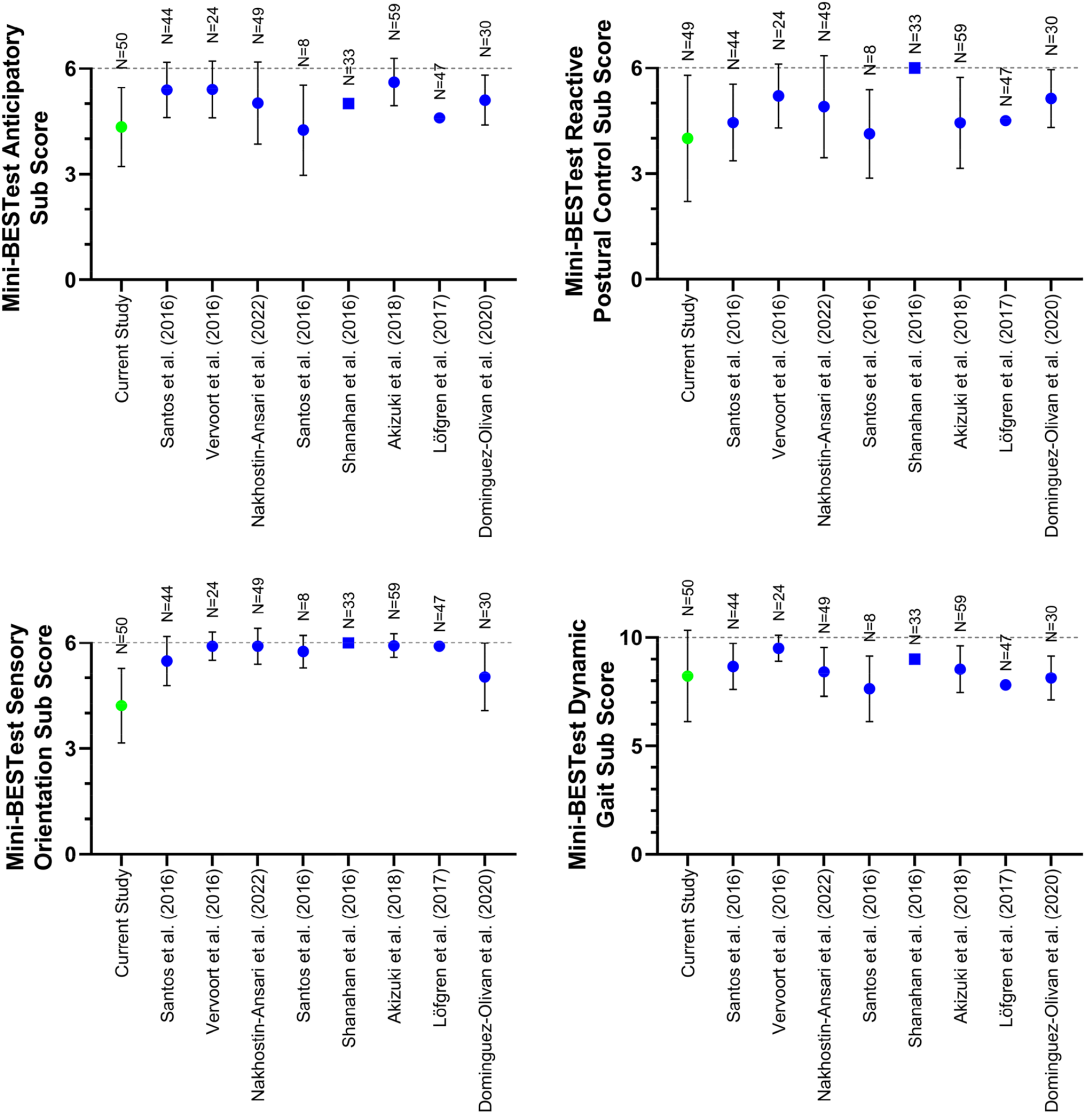
Mini-BESTest sub scores (circles: mean ± standard deviation; squares: medians) for the current participants with BVP and healthy reference values found in the literature (left to right is youngest to oldest mean age). Values are shown here as originally reported, meaning that different rounding conventions have been used

Within the BVP group, the Friedman test revealed a significant sub score effect (*χ*^2^_F_ (3) = 29.86, *p* < 0.001) and post hoc Wilcoxon signed rank tests revealed significant differences between the relative dynamic gait sub score and all other sub scores (*p* < 0.001). No other sub score comparisons were statistically significant (anticipatory vs. reactive postural control: *p* = 0.11; anticipatory vs. sensory orientation: *p* = 0.22; reactive postural control vs. sensory orientation: *p* = 0.38). Mann–Whitney *U* tests revealed that, compared to the healthy control reference data (Fig. 3), the scores of the participants with BVP were significantly lower for anticipatory, reactive postural control and sensory orientation sub scores [*U*(*N*_BVP_ = 50, *N*_Healthy_ = 190) = 2364.50, *p* < 0.001, *d* = 0.75;* U*(*N*_BVP_ = 50, *N*_Healthy_ = 190) = 3737, *p* = 0.028, *d* = 0.3; and *U*(*N*_BVP_ = 50, *N*_Healthy_ = 190) = 1223.50, *p* < 0.001, *d* = 1.22, respectively], while the dynamic gait sub scores were not statistically different [*U*(*N*_BVP_ = 50, *N*_Healthy_ = 190) = 4374.50, *p* = 0.367, *d* = 0.11]. Participants with BVP scored (mean ± SD) 4.34 ± 1.12, 4.00 ± 1.79, 4.22 ± 1.96 and 8.22 ± 2.11 and the healthy controls scored 5.27 ± 0.93, 4.66 ± 1.25, 5.66 ± 0.68 and 8.43 ± 1.10 for anticipatory, reactive postural control, sensory orientation and dynamic gait sub scores, respectively. Note that the reactive postural control score, while statistically significant, did not exceed the effect size for which our analysis was powered and should be interpreted cautiously. In this analysis, the sensory orientation sub score showed the largest effect, and this is also supported by visual comparison with the literature values in Fig. 4, which does support our expectation that sub scores more directly related to sensory function would show larger deficits.

### Mini-BESTest total scores and falls history

In the 49 participants with BVP with Mini-BESTest total scores, 25 did not report falling (Mini-BESTest Mean ± SD, Median: 21.4 ± 5.8, 23) and 24 did report falling (20.1 ± 5.01, 21), 18 with two or more falls and six with a single fall. A Mann–Whitney U tests did not find a statistically significant difference in the Mini-BESTest total score between these groups [*U*(*N*_nofalls_ = 25, *N*_falls_ = 24) = 242, *p* = 0.249, *d* = 0.235].

### Exploratory analyses on the age-balance association

**Fig. 5:**
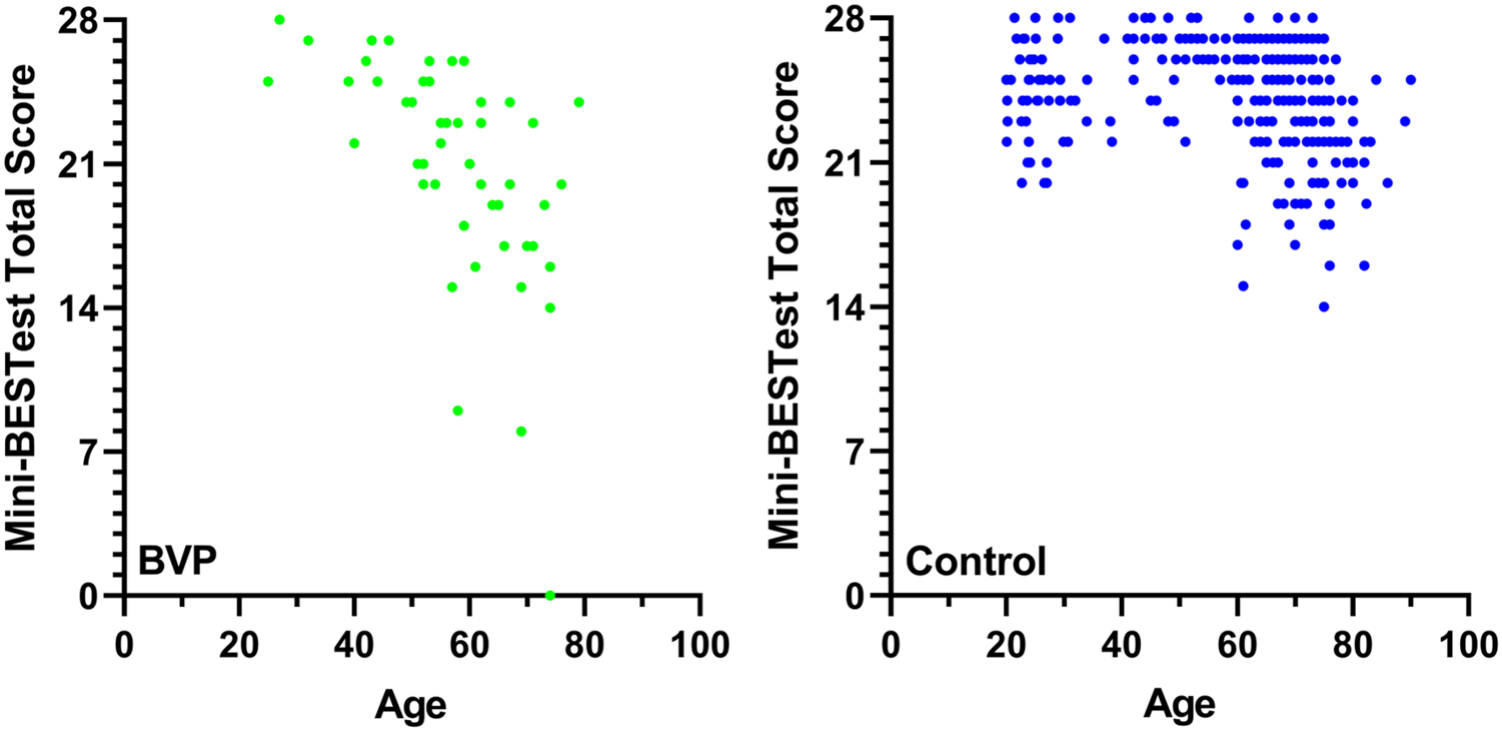
Scatter plots indicating the relationship between age and Mini-BESTest total score in BVP and healthy control groups

**Fig. 6:**
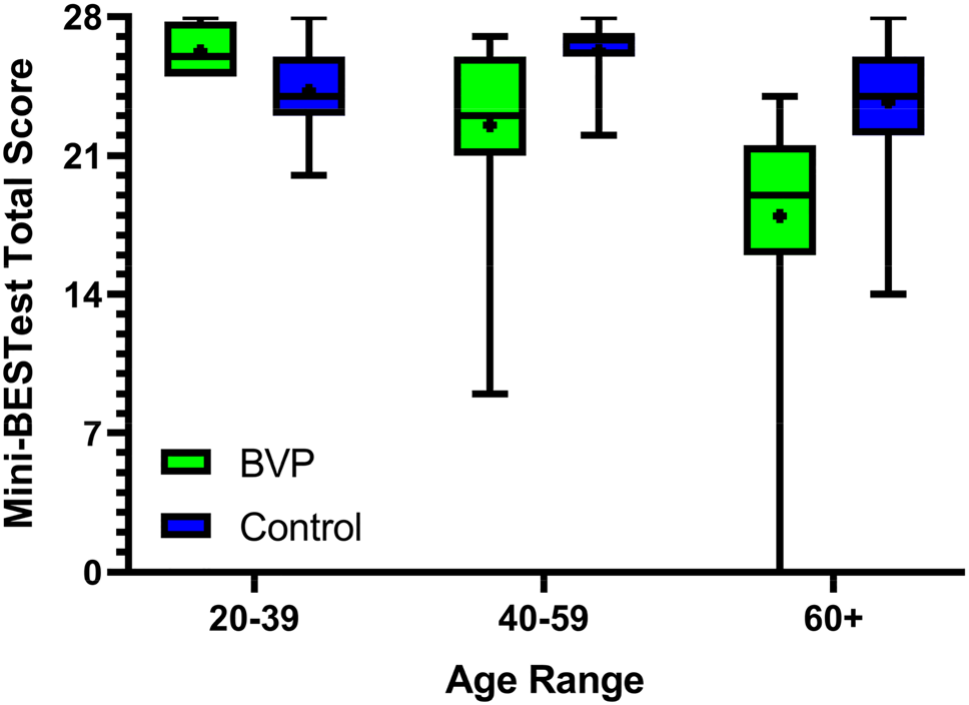
Mini-BESTest total score across young (20–39 years), middle-aged (40–59 years) and older (60+ years) adult age groups in our participants with BVP and healthy control data from the literature. The boxplots indicate the medians, interquartile range and minimum and maximum values, with the crosses indicating the mean values

In our data from the participants with BVP, we observed a potential age effect on the Mini-BESTest scores. To further explore this, we conducted Spearman correlations between participant age and Mini-BESTest total scores for the BVP and healthy control data separately. For the participants with BVP, a significant negative correlation between Mini-BESTest total score and age was found [Spearman's correlation coefficient = − 0.67 (95% CI − 0.74 to − 0.35), *p* < 0.001], and for the healthy control group a significant negative correlation was also found [Spearman's correlation coefficient = − 0.32 (95% CI − 0.32 to − 0.11, *p* < 0.001]. Scatter plots of these data can be seen in Fig. 5. This difference in age effect is also visible when visualizing the Mini-BESTest total scores separated into young (20–39 years), middle-aged (40–59 years) and older (60 + years) adult age groups (Fig. 6) and when comparing sub score data across these age groups (Table 3).

**Table 3:** Age group Mini-BESTest total and sub scores (mean ± SD) for the obtained data in BVP and healthy control groups

Variables	Age group	BVP	*N*	Control	*N*
Total score (maximum: 28)	(20–39)	26.25 ± 1.50	4	24.28 ± 2.21	50
	(40–59)	22.52 ± 4.21	23	26.26 ± 1.43	42
	(60 +)	17.95 ± 5.53	22	23.69 ± 2.74	213
Anticipatory (maximum 6)	(20–39)	5.50 ± 0.58	4	5.37 ± 0.80	46
	(40–59)	4.71 ± 0.86	24	5.85 ± 0.49	20
	(60+)	3.73 ± 1.12	22	5.14 ± 0.99	124
Reactive postural control (maximum 6)	(20–39)	5.50 ± 0.58	4	4.52 ± 1.11	46
	(40–59)	4.52 ± 1.70	23	5.40 ± 0.94	20
	(60+)	3.18 ± 1.68	22	4.59 ± 1.31	124
Sensory orientation (maximum 6)	(20–39)	5.75 ± 0.50	4	5.50 ± 0.69	46
	(40–59)	4.38 ± 0.82	24	6.00 ± 0.00	20
	(60+)	3.77 ± 1.07	22	5.67 ± 0.72	124
Dynamic gait (maximum 10)	(20–39)	9.50 ± 0.58	4	8.65 ± 1.08	46
	(40–59)	8.88 ± 1.51	24	9.05 ± 0.69	20
	(60+)	7.27 ± 2.47	22	8.24 ± 1.12	124

## Discussion

We aimed to determine whether the Mini-BESTest can be used with people with BVP and to explore how people with BVP perform in terms of their overall score and their subcomponent scores and if these reveal consistent balance deficits within this group and in relation to healthy reference data. Our findings supported our hypothesis that BVP would perform significantly worse than healthy controls on the Mini-BESTest total score. Regarding the sub scores, our expectation that subcomponents with more sensory disturbance would reveal larger deficits was partly supported, since the sensory orientation sub score was significantly lower in BVP compared to the healthy control data and the effect size of this difference was the largest difference of the sub score comparisons. However, when compared to the other sub scores within the BVP group, the sensory orientation was only significantly lower than the dynamic gait sub score.

Regarding the application and use of the Mini-BESTest in the BVP population, 49 of our 50 participants had complete scores. The reactive postural control part of the test could not be completed for one participant for practical reasons unrelated to the patient’s ability. Floor and ceiling effects were not observed, as only one patient (2.04%) scored 0 and only one patient (2.04%) scored the maximum score which is similar to previous studies on mixed balance disorders [49], Parkinson’s disease [50] and chronic stroke [51]. We experienced no other issues related to feasibility or safety when performing the test. As a result, we conclude that the Mini-BESTest can be used in the BVP population, as has been shown with other patient populations such as people with balance disorders [49], Parkinson’s disease [50, 52], chronic stroke [51] and subacute stroke [53]. While feasible, there is currently no data on the reliability of the Mini-BESTest in the BVP population, which future work should address.

The Mini-BESTest scores in our participants with BVP (mean ± SD: 20.78 ± 5.41, median: 22, interquartile range: 19.0–25.0) are similar to those in some previous reports in people with chronic stroke [51] (*n* = 106, median: 19.0, interquartile range: 14.0–22.0) and Parkinson’s disease [50, 52] (King et al. [50]: *n* = 97, median: 23, interquartile range: 20.0–24.0, mean ± SD: 21.8 ± 3.6; Leddy et al. [52]: *n* = 80, Mean ± SD: 20.2 ± 7.0). Our participants with BVP were significantly taller and heavier, with a higher BMI than the healthy control group which may in part be due to the Netherlands being a taller than average population, combined with the negative effects on daily life physical activity due to BVP [15]. We cannot clearly determine the potential role these differences might have in the Mini-BESTest performance with the current data. However, the age-Mini-BESTest total score association results did not differ much when we reanalysed the data with a partial Spearman correlation with BMI as a control variable (BVP: − 0.62, *p* < 0.001; control: − 0.27, *p* < 0.001), suggesting that this difference is not a major contributing factor. Finally, we did not find significant differences in Mini-BESTest total scores between participants with BVP with and without falls in the previous 12 months, despite previous reports that the Mini-BESTest can significantly distinguish between people with and without falls history in other populations [42, 51, 52, 54]. The percentage of participants with BVP with a recent history of falls (48%) was slightly higher than the literature average of 42% reported in a previous review on falls in BVP [4]. While not the main purpose of this study, we also performed area under the receiver operator characteristic curve and sensitivity and specificity analyses for Mini-BESTest total and sub scores and these results can be found in the supplement (Online Resource 1).

We observed a significant association between age and Mini-BESTest total score in both BVP and healthy groups. One of the included studies with healthy control data has previously reported an age-balance relationship [38]. The association was much stronger in the BVP group which we suggest may be related to less possibility for sensory compensation and re-weighting with increasing age. Aside from the vestibular system, proprioception and vision are important for balance control and all show a gradual age-related decline [25, 55–57] and previous experimental studies have demonstrated more substantial balance performance decline when multiple sensory systems are perturbed [20, 58–61]. These findings imply that close attention might need to be paid to proprioceptive and visual system health in people with BVP to identify and help patients with less possibility for sensory compensation. Sensory substitution devices may also have a role to play here [62].

The current study has some limitations that should be kept in mind. Since participants in this study were volunteering to participate in a full day of assessments as part of the larger study, this may have influenced who chose to participate. We did not record physical activity, sport or physical therapy or rehabilitation history, so prior experience with balance tasks and training may have varied across the participants. One important consideration is that, while sub scores of the Mini-BESTest have been analysed here, these sub scores are not necessarily independent and significant correlation between the sub scores and tasks may exist [63]. While we have reported sub score-specific differences, these should be interpreted with caution and should not necessarily be used for clinical decision making. One participant behaved nervously and did not appear to perform the tasks correctly for psychosomatic reasons (note that this was not the participant that scored 0). Since we had no a priori criteria for how do deal with this situation, we included this participant in the analysis, but also repeated the analysis excluding the participant to examine if this individual score would have an effect on the overall results (this did not lead to any changes in the statistical significance outcomes of the tests, the direction of effects or the conclusions).

In conclusion, our findings demonstrate that the Mini-BESTest can feasibly be used in people with BVP. The Mini-BESTest reveals significantly reduced balance performance in people with BVP compared to healthy control reference data. Within both BVP and healthy groups, a significant negative association between balance performance and age was found, with the association being twice has high in BVP, indicating that reduced capacity for sensory compensation with increasing age might need to be considered in the assessment and care of people with BVP.

## Supplementary Information

Below is the link to the electronic supplementary material.

Supplementary file1 (PDF 371 KB)

## Data Availability

Anonymised data of the participants with BVP (some demographic data excluded) and the R code for the statistical analysis performed in Jamovi can be found at: http://www.doi.org/10.17605/OSF.IO/TFKN3 The healthy control data obtained from the literature are not publicly shared since the authors of this article are not the owners of that data, but they are available on request.
