# Supplementary material for: Assessing balance in people with bilateral vestibulopathy using the Mini-Balance Evaluation Systems Test (Mini-BESTest): feasibility and comparison with healthy control data
Source: J Neurol. 2023 Jun 3;270(9):4423–33. doi: 10.1007/s00415-023-11795-y (PMC10421784; doi:10.1007/s00415-023-11795-y)
Supplement: Supplementary file 1 — Supplementary file1 (PDF 371 KB) [file 415_2023_11795_MOESM1_ESM.pdf]

Supplementary Information for:

Assessing Balance in People with Bilateral Vestibulopathy using the Mini-Balance Evaluation Systems Test (Mini-BESTest): Feasibility and Comparison with Healthy Control Data

Journal of Neurology

Meichan Zhu[1,2,3*], Lisa van Stiphout[1], Mustafa Karabulut[1], Angélica Pérez Fornos[4], Nils Guinand[4], Kenneth Meijer[2], Raymond van de Berg[1], Christopher McCrum[2*]

[1]Department of Otorhinolaryngology and Head and Neck Surgery, Division of Balance Disorders, Maastricht University Medical Center, School for Mental Health and Neuroscience, Maastricht, The Netherlands
[2]Department of Nutrition and Movement Sciences, NUTRIM School of Nutrition and Translational Research in Metabolism, Maastricht University, Maastricht, The Netherlands
[3]Department of Otorhinolaryngology, Guangzhou Twelfth People's Hospital (Guangzhou Otolarynology- head and Neck Surgery hospital), No1.Tianqiang Road, Tianhe District, Guangzhou, Guangdong,510620, China
[4]Service of Otorhinolaryngology and Head and Neck Surgery, Department of Clinical Neurosciences, Geneva University Hospitals, Geneva, Switzerland

*Correspondence:
Meichan Zhu: z.meichan@maastrichtuniversity.nl
Christopher McCrum: chris.mccrum@maastrichtuniversity.nl

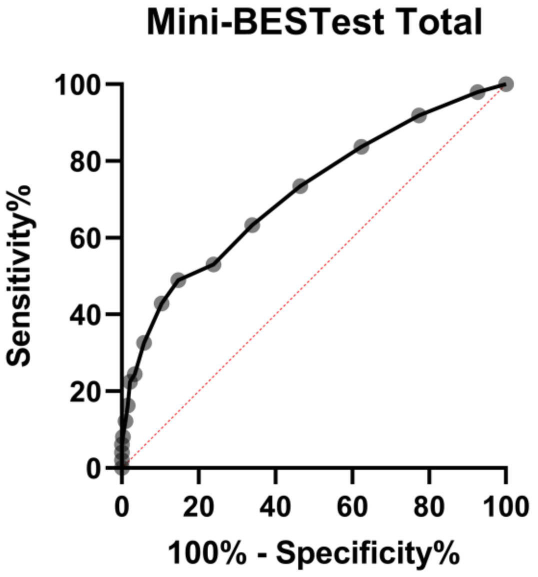

Supplementary Figure 1: Receiver operator characteristic (ROC) curve for prediction of BVP based on the Mini-BESTest total score.

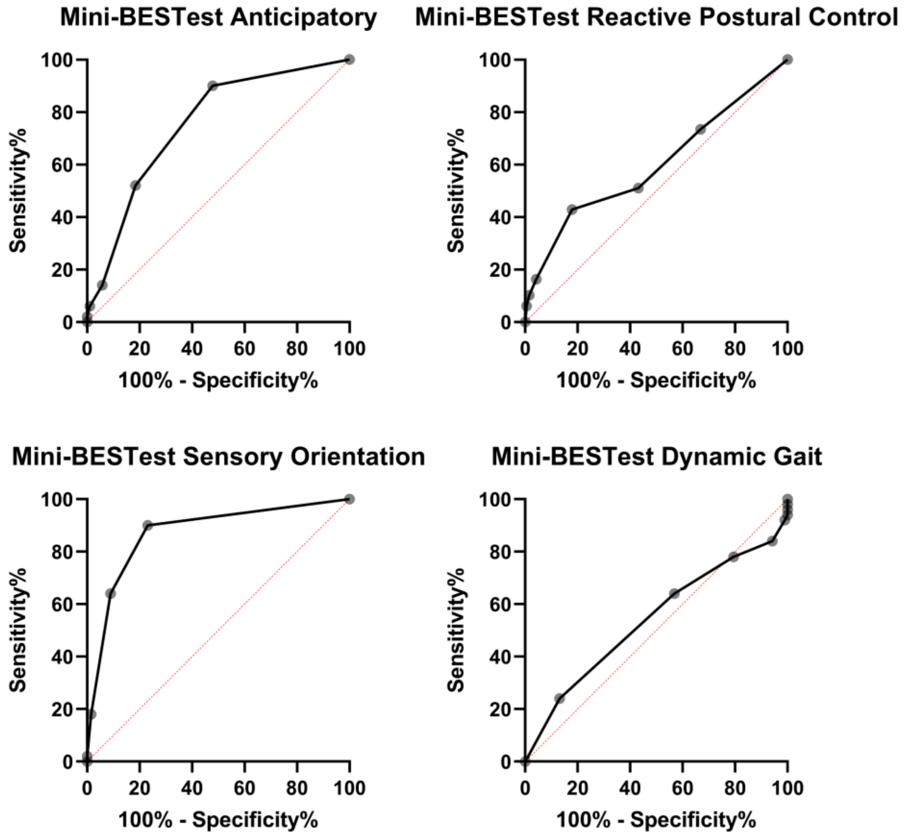

Supplementary Figure 2: Receiver operator characteristic (ROC) curves for prediction of BVP based on the Mini-BESTest sub scores.

Supplementary Table 1: Receiver Operator Characteristic Curve Outcomes for the Mini-BESTest Total and Sub Scores

Area under the ROC curve	Mini-BEST Total	Anticipatory	Reactive Postural Control	Sensory Orientation	Dynamic Gait
Area	0.72	0.75	0.60	0.87	0.54
Std. Error	0.043	0.036	0.050	0.030	0.050
95% CI	0.63 to 0.80	0.68 to 0.82	0.50 to 0.70	0.81 to 0.93	0.44 to 0.64
P value	<0.0001	<0.0001	0.0334	<0.0001	0.3900
Controls	327	190	190	190	190
Patients	49	50	49	50	50

Supplementary Table 2: Sensitivity and Specificity Outcomes for the Mini-BESTest Total Score

Cut-off	Sensitivity %	95% CI	Specificity %	95% CI	Likelihood ratio
< **4.000**	2.041	0.1047% to 10.69%	100.0	98.84% to 100.0%	
< **8.500**	4.082	0.7252% to 13.71%	100.0	98.84% to 100.0%	
< **11.50**	6.122	2.104% to 16.52%	100.0	98.84% to 100.0%	
< **14.50**	8.163	3.220% to 19.19%	99.69	98.29% to 99.98%	26.69
< **15.50**	12.24	5.735% to 24.24%	99.08	97.34% to 99.75%	13.35
< **16.50**	16.33	8.513% to 29.04%	98.47	96.47% to 99.35%	10.68
< **17.50**	22.45	13.02% to 35.88%	97.86	95.65% to 98.96%	10.49
< **18.50**	24.49	14.60% to 38.09%	96.64	94.08% to 98.11%	7.280
< **19.50**	32.65	21.21% to 46.62%	94.19	91.10% to 96.25%	5.620
< **20.50**	42.86	30.02% to 56.73%	89.60	85.82% to 92.46%	4.122
< **21.50**	48.98	35.58% to 62.53%	85.32	81.08% to 88.75%	3.337
< **22.50**	53.06	39.38% to 66.30%	76.15	71.24% to 80.45%	2.224
< **23.50**	63.27	49.27% to 75.33%	66.06	60.76% to 70.97%	1.864
< **24.50**	73.47	59.74% to 83.79%	53.52	48.10% to 58.85%	1.581
< **25.50**	83.67	70.96% to 91.49%	37.61	32.54% to 42.98%	1.341
< **26.50**	91.84	80.81% to 96.78%	22.63	18.43% to 27.47%	1.187
< **27.50**	97.96	89.31% to 99.90%	7.339	4.981% to 10.69%	1.057

Supplementary Table 3: Sensitivity and Specificity Outcomes for the Mini-BESTest Sub Scores

Sub Score	Cut-off	Sensitivity %	95% CI	Specificity %	95% CI	Likelihood ratio
**Anticipatory**	< 1.000	2.000	0.1026% to 10.50%	100.0	98.02% to 100.0%	
	< 2.500	6.000	1.635% to 16.22%	98.95	96.24% to 99.81%	5.700
	< 3.500	14.00	6.951% to 26.19%	94.21	89.93% to 96.74%	2.418
	< 4.500	52.00	38.51% to 65.20%	81.58	75.46% to 86.45%	2.823
	< 5.500	90.00	78.64% to 95.65%	52.11	45.03% to 59.10%	1.879
**Reactive Postural Control**	< 0.5000	6.122	2.104% to 16.52%	99.47	97.08% to 99.97%	11.63
	< 1.500	10.20	4.438% to 21.76%	98.42	95.46% to 99.57%	6.463
	< 2.500	16.33	8.513% to 29.04%	95.79	91.91% to 97.85%	3.878
	< 3.500	42.86	30.02% to 56.73%	82.11	76.04% to 86.90%	2.395
	< 4.500	51.02	37.47% to 64.42%	56.84	49.73% to 63.68%	1.182
	< 5.500	73.47	59.74% to 83.79%	33.16	26.86% to 40.13%	1.099
**Sensory Orientation**	< 1.500	2.000	0.1026% to 10.50%	100.0	98.02% to 100.0%	
	< 3.500	18.00	9.770% to 30.80%	98.42	95.46% to 99.57%	11.40
	< 4.500	64.00	50.14% to 75.86%	91.05	86.14% to 94.34%	7.153
	< 5.500	90.00	78.64% to 95.65%	76.84	70.35% to 82.27%	3.886
**Dynamic Gait**	> 1.000	98.00	89.50% to 99.90%	0.000	0.000% to 1.982%	0.9800
	> 2.500	96.00	86.54% to 99.29%	0.000	0.000% to 1.982%	0.9600
	> 4.000	94.00	83.78% to 98.36%	0.000	0.000% to 1.982%	0.9400
	> 5.500	92.00	81.16% to 96.85%	1.053	0.1870% to 3.756%	0.9298
	> 6.500	84.00	71.49% to 91.66%	5.789	3.263% to 10.07%	0.8916
	> 7.500	78.00	64.76% to 87.25%	20.53	15.39% to 26.83%	0.9815
	> 8.500	64.00	50.14% to 75.86%	43.16	36.32% to 50.27%	1.126
	> 9.500	24.00	14.30% to 37.41%	86.84	81.30% to 90.93%	1.824
